# Edge AI Model Deployed for Real-Time Detection of Atrial Fibrillation Risk during Sinus Rhythm

**DOI:** 10.3390/jcm13082218

**Published:** 2024-04-11

**Authors:** Hongmin Wu, Takumi Sawada, Takafumi Goto, Tatsuya Yoneyama, Tetsuo Sasano, Ken Asada

**Affiliations:** 1 Technology & Innovation Department, Fukuda Denshi Co., Ltd., Tokyo 113-8420, Japan; 2 Development Headquarters, Fukuda Denshi Co., Ltd., Tokyo 113-8420, Japan; 3 Department of Cardiovascular Medicine, Tokyo Medical and Dental University, Tokyo 113-8519, Japan; 4 Cancer Translational Research Team, RIKEN Center for Advanced Intelligence Project, Tokyo 103-0027, Japan; 5 Division of Medical AI Research and Development, National Cancer Center Research Institute, Tokyo 104-0045, Japan

**Keywords:** atrial fibrillation, sinus rhythm, standard 12-lead ECGs, deep learning-based, edge AI deployment

## Abstract

**Objectives:** The study aimed to develop a deep learning-based edge AI model deployed on electrocardiograph (ECG) devices for the real-time detection of atrial fibrillation (AF) risk during sinus rhythm (SR) using standard 10 s, 12-lead electrocardiograms (ECGs). **Methods:** A novel approach was used to convert standard 12-lead ECGs into binary images for model input, and a lightweight convolutional neural network (CNN)-based model was trained using data collected by the Japan Agency for Medical and Research Development (AMED) between 2019 and 2022. Patients over 40 years old with digital, SR ECGs were retrospectively enrolled and divided into AF and non-AF groups. The data labeling was supervised by cardiologists. The dataset was randomly allocated into training, validation, and internal testing datasets. External testing was conducted on data collected from other hospitals. **Results:** The best-trained model achieved an AUC of 0.82 and 0.80, sensitivity of 79.5% and 72.3%, specificity of 77.8% and 77.7%, precision of 78.2% and 76.4%, and overall accuracy of 78.6% and 75.0% in the internal and external testing datasets, respectively. The deployed model and app package utilized 2.5 MB and 40 MB of the available ROM and RAM capacity on the edge ECG device, correspondingly. The processing time for AF risk detection was approximately 2 s. **Conclusions:** The model maintains comparable performance and improves its suitability for deployment on resource-constrained ECG devices, thereby expanding its potential impact to a wide range of healthcare settings. Its successful deployment enables real-time AF risk detection during SR, allowing for timely intervention to prevent AF-related serious consequences like stroke and premature death.

## 1. Introduction

AF is a common arrhythmia, with an estimated prevalence of 3% in adults [1], and is associated with an elevated risk of stroke, heart failure, and premature death [2]. However, the early detection of AF, particularly paroxysmal AF, is very challenging due to its asymptomatic or infrequent nature. Even when patients present with symptoms such as palpitations or chest discomfort, standard ECG examinations often show SR. Some studies suggest that the progression of AF can induce electrical and structural changes, manifesting as subtle patterns on normal SR ECGs [3]. However, currently, it remains difficult for cardiologists to manually distinguish AF on ECGs with normal SR.

With the rapid progress and breakthroughs brought about by artificial intelligence (AI) technology, serval studies demonstrated that some subtle signals caused by clinically important phenomena can be detected with AI in ECG data that are imperceptible to the human eye [4]. Some studies have reported promising results from well-trained AI models in extracting relevant features from subtle pattern changes in 12-lead ECGs [3,5,6]. However, these studies often encountered imbalanced datasets between the positive and negative classes. Furthermore, each patient had unequal numbers of ECG records included in training, validation, and testing datasets, which could potentially mislead the prediction accuracy and the estimated area under the receiver operating characteristic (ROC) curve (AUC) [7,8]. Variations exist among various studies, particularly in four key aspects: dataset composition and pre-processing, types of model input, deep learning model architectures, and classification approaches [9]. Despite these advancements, much of the existing literature remains confined to academic research and lacks exploration into the feasibility and efficiency of methods for deploying edge AI.

In this study, we proposed a novel approach to convert standard 10 s, 12-lead ECGs into binary images for model input and designed a lightweight CNN model to enable real-time AF risk detection on edge ECG devices. The dataset was well balanced between the AF and non-AF groups, with each patient contributing an equal amount of ECG data, specifically one ECG datum per patient for testing. Performance evaluation and statistical analysis were conducted using internal and external testing datasets collected from diverse clinical facilities in Japan.

## 2. Methods

### 2.1. Ethics and Data Collection

Approval for data collection was obtained from the Ethics Committees of the Tokyo Medical and Dental University. A total of 3109 ECGs from 2930 patients aged over 40 years were retrospectively collected from seven affiliated hospitals between September 2019 and March 2022. The study adhered to the Code of Ethics of the World Medical Association (Declaration of Helsinki) and the Ethical Guidelines for Medical and Health Research Involving Human Subjects issued by the Ministry of Education of Japan in 2015. Only data from individuals who provided consent were used, and all records were anonymized. All ECGs were recorded at a sampling rate of 500 Hz with a 10 s length using FCP-8800 ECG machines manufactured by Fukuda Denshi, Tokyo, Japan. Diagnostic labels were assigned by trained physicians under the supervision of cardiologists.

**Figure 1:**
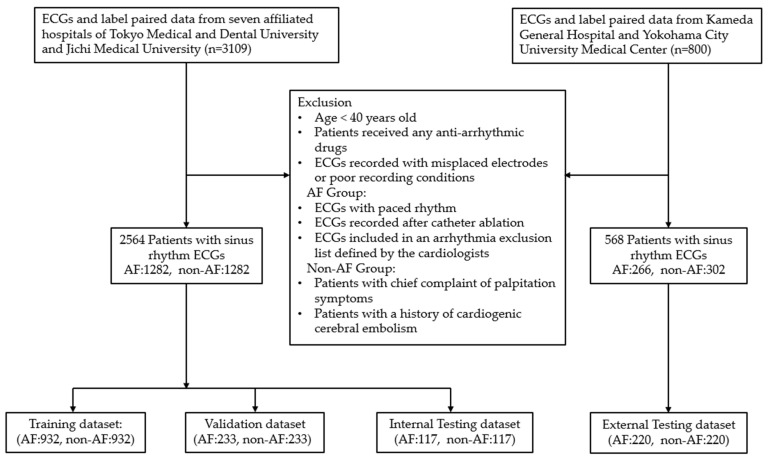
The flowchart of data collection and dataset composition. A total of 3909 ECGs from 3730 patients were collected for training, validation, internal testing, and external testing. Following the exclusion criteria, SR ECGs collected from 1282 patients with AF and 1282 patients from the control group were randomly allocated into three datasets for training, validation, and internal testing. Additionally, an external testing dataset comprised 440 SR ECGs collected from 220 patients with AF and 220 patients from the control group.

The flowchart detailing the data collection and data composition is presented in Figure 1. A total of 1668 ECGs from 1489 patients with AF records and 1441 ECGs from 1441 non-AF patients were initially collected. After applying exclusion criteria and selecting one 12-lead SR ECG datum per patient, from both groups, three datasets were prepared: a training and validation dataset with a ratio of 8:2, comprising 2330 ECGs (AF: 1165, non-AF: 1165). The remaining 234 ECGs (AF: 117, non-AF: 117) were used as the internal testing dataset.

Additionally, to assess the generalization ability and external performance validation of AF risk detection, 800 (AF: 400, non-AF: 400) more ECGs with paired label data were retrospectively collected from Kameda General Hospital and Yokohama City University Medical Center from April 2023 to July 2023. Approval for data collection was obtained from the Ethics Committees of these two facilities. All ECG records were anonymized and an opt-out form on a website was used as an acceptable method to obtain consent from the patients. These two distinct facilities did not contribute any data to the model training. The same SR ECG inclusion and exclusion criteria described above were applied for data selection. According to the determined sample size for performance validation, a total of 220 ECGs from 220 patients in the AF group were randomly selected, and an equal number of SR ECGs with similar patient characteristics were matched from the non-AF group, resulting in an external testing dataset comprising 440 ECGs (AF: 220, non-AF: 220).

### 2.2. Identifying Study Groups and Selecting SR ECGs

Both the digital SR ECGs and the extracted labels of the included patients were collected. The dataset was divided into two groups: one group labeled as AF, consisting of patients with at least one documented AF episode within the past 2 years before the collected SR ECGs, and the other labeled as non-AF, consisting of patients without any chief complaint of palpitation symptoms and without an AF diagnostic code in their electronic medical records. Patients with an AF diagnostic code but no corresponding ECG documentation of AF were excluded from the performance analysis to mitigate ambiguity.

**Figure 2:**
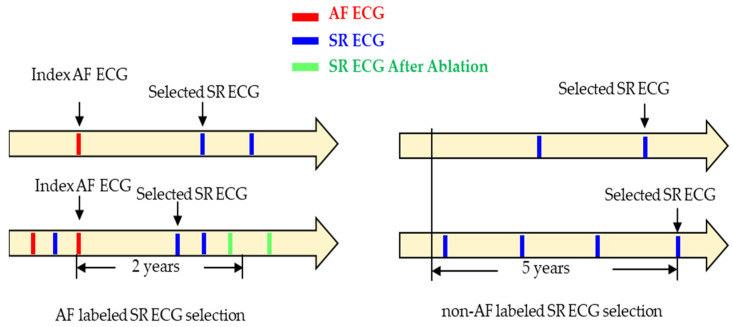
The selection process of SR ECGs in both the AF group and non-AF group. The top and bottom two examples depict the SR ECG selection for the two groups, respectively.

The inclusion criteria for selecting SR ECGs in both groups are illustrated in Figure 2. For the AF group, the last event of AF ECG served as an index, and SR ECGs within 2 years following this index were considered for selection. If multiple SR ECGs were available, the one closest to the index within the 2-year window was selected. SR ECGs recorded before the index or after catheter ablation were excluded. The figure on the right side illustrates examples of SR ECG selection in the non-AF group. The latest SR ECG served as an index. The window of interest was defined as a timeframe of 5 years before the index SR ECG. If there was a presence of at least one more SR ECG before it within the 5-year period, the SR ECG was selected. Otherwise, it was discarded.

According to the prevalence analysis of atrial fibrillation in the general population of Japan [10,11], all patients included in both study groups were required to be over 40 years old at the time the selected SR ECG was recorded. Additionally, none of the patients in either group received any anti-arrhythmic drugs. The following six criteria were applied for data exclusion: (1) ECGs with paced rhythms. (2) ECGs recorded after catheter ablation or heart surgery. (3) Patients with mitral stenosis or artificial valve replacement. (4) Patients with a history of cardiogenic cerebral embolism in the control group. (5) ECGs included in an arrhythmia exclusion list defined by the cardiologists (Supplementary Materials, Table S1). (6) ECGs recorded with misplaced electrodes or poor recording conditions.

### 2.3. Data Pre-Processing and Model Input Type

ECG signals often contain various types of noises and artifacts, such as power line interference, myoelectric noise, base-line drift, and high-frequency noise components that arise from the device or environment. The corresponding digital filters are provided on the ECG device. Clinicians may apply different filters during ECG recording to remove noise, and information about the applied filters is recorded in the saved ECG data. To standardize the conditions of all collected ECGs, the unused filters among the provided four filters were applied to the ECG signals for the uniform noise removal of all ECGs.

**Figure 3:**
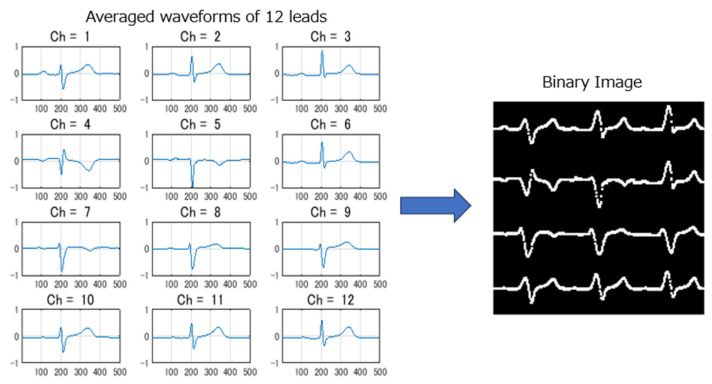
Converting the 12 averaged waveforms into a composite binary image.

Since all ECGs were collected during SR for 10 s and with 12 leads, the data dimensionality was high for 12-lead ECG signals. Some researchers used only a subset of 12 leads or part of signal segments to reduce the computation cost, but still, quite a deep AI model needs to be used for good performance. This increases the difficulty of high memory usage for edge AI deployment on resource-constrained devices. In this study, we proposed a novel approach to transform standard 10 s, 12-lead ECGs into binary images for model input and to design a lightweight CNN model for real-time AF risk detection on edge ECG devices. Five steps of signal pre-processing were conducted: (1) An R wave-triggered signal averaging method was used to generate averaged ECGs with a length of 1 s for each lead. (2) The averaged waveforms were compressed along the time and amplitude axes to an appropriate size suitable for deployment. (3) The compressed averaged waveform was converted into a binary image using brightness processing. (4) Binary images from 12 leads were arranged into a composite image with a layout of 4 rows and 3 columns. (5) The total image resolution was adjusted to align with the depth of the CNN model and suitable for deployment. The selection of image resolution is intricately linked to the model’s depth: higher resolutions necessitate deeper models for optimal performance. However, constraints on ROM and RAM capacity in edge devices limit model size and processing time. Therefore, achieving a balance between image resolution and model performance is crucial. We conducted tests with various image resolutions and corresponding models, ultimately selecting a compromised resolution. Further detailed investigation into optimal resolution settings will be explored in future work. Figure 3 illustrates an example of the 12 generated average waveforms being converted into a composite binary image.

### 2.4. Model Architecture and Deployment

**Figure 4:**
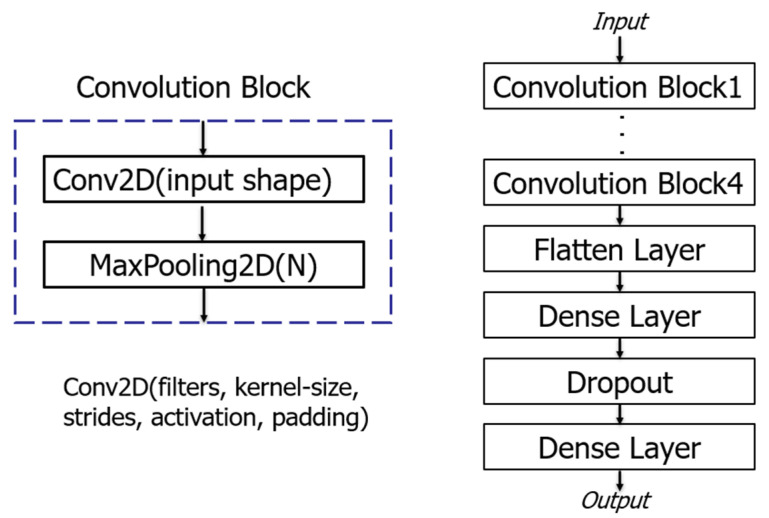
The architecture of the AI model.

To ensure that the AI model remains compact, efficient, and accurate, a convolutional neural network with a small number of layers was implemented using the Keras package with a TensorFlow backend in Python (v3.7). The architecture of the model consisted of four convolution blocks, each comprising a two-dimensional convolution layer with a kernel size of 3 × 3, ReLU activation function, 64 different filters, and a max-pooling layer as illustrated in Figure 4. After the final max-pooling layer, the extracted ECG features were input into a fully connected layer (Flatten layer), two dense layers, and a dropout layer, before being fed into an output layer activated with softmax function for AF classification. The batch size was set to 64, and 150 epochs, an Adam optimizer, and a categorical cross-entropy loss function were employed to iteratively update network weights trained on a computer equipped with an NVIDIA GeForce GTX1080 GPU (8 GB), sold by Tokyo Computer Service Co. Ltd., Tokyo, Japan. The initial learning rate was set to 0.0001 with a learning rate decay of 1 × 10 ^−6^.

The trained AI model, developed in the Python environment, was saved in the JSON format and uploaded, along with the necessary header library, into the app package written in C++ for on-board AF risk detection. The model was optimized to ensure low memory usage, making it suitable for deployment on resource-constrained ECG devices.

### 2.5. Outcome Assessments

Performance metrics refer to mathematical formulas that are used for assessing how well an AI model predicts clinical or other health outcomes from the data. In binary classification tasks, where outcomes are classified into two categories, several metrics such as accuracy, sensitivity, specificity, precision, and AUC are commonly used. While accuracy and AUC are suitable for well-balanced datasets, they may not be appropriate for datasets with class imbalances. To address potential bias and ensure robust evaluation, all datasets in this study, including those used for training, validation, internal testing, and external testing, were well balanced between AF and non-AF groups. This allows for the comprehensive assessment of model performance using all the metrics mentioned above.

### 2.6. Statistical Analysis

The statistical analysis involves collecting and analyzing large volumes of data to identify trends and develop insights. Once the final fitted model was obtained, a statistical analysis plan was designed and specified in advance for external testing. The plan included the following steps: (1) The descriptive and inferential analysis of the clinical characteristics of patients included in the AF and non-AF groups. The mean, standard deviation, and independent *t*-tests for continuous variables, and percentages and Fisher exact tests for categorical variables were calculated and performed to verify if there were statistically significant differences (*p* < 0.05) in clinical variables between the patients in the two groups. (2) The measurement of outcomes and the estimation of their 95% confidence intervals. (3) Special data testing for non-AF identification. All the statistical analyses were performed using EZR version 1.55 and R version 4.3.1 software.

## 3. Results

### 3.1. Internal Testing

The model was input with the binary ECG images and trained using the dataset (*n* = 2330, AF: 1165, non-AF: 1165) as described in Section 2.1. To enrich the training dataset, a representative waveform, termed the dominant waveform, was extracted from each lead of the recorded 10 s, 12-lead ECGs. These dominant waveforms exhibited less noise and matched the 1 s length of the averaged waveforms used for training. Following the signal pre-processing described in Section 2.3, two binary ECG images were generated per patient, effectively doubling the dataset size to *n* = 4660 (AF: 2330, non-AF: 2330) for model training.

**Figure 5:**
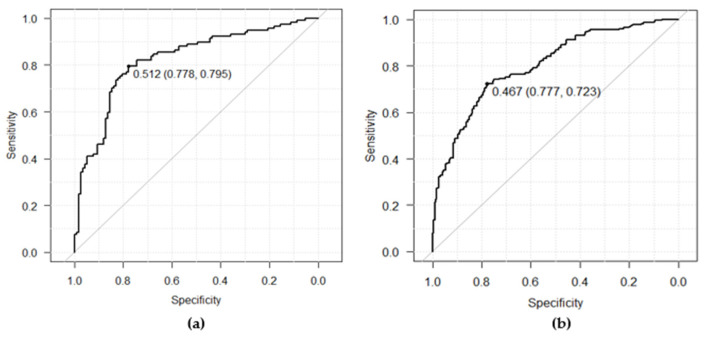
The ROC curves: (**a**) on the internal testing dataset; (**b**) on the external testing dataset.

For internal testing, a dataset comprising 234 ECGs (AF: 117, non-AF: 117) was utilized. Each patient contributed one binary image using the averaged waveforms, and no dominant ECG waveforms were used for testing. The outcome metrics of the internal testing were measured as follows: AUC, 0.82 (95% CI 0.77–0.88); sensitivity, 79.5% (95% CI 71.0–86.4); specificity, 77.8% (95% CI 69.2–84.9); precision, 78.2% (95% CI 69.6–85.2); and accuracy, 78.6% (95% CI 72.8–83.7). The ROC curve was depicted on the left side in Figure 5a to compare with the ROC curve shown in Figure 5b obtained from the following external testing in Section 3.2.4.

### 3.2. External Testing

#### 3.2.1. External Dataset Analysis

The external testing and statistical analysis were further conducted using the external dataset (*n* = 440, AF: 220, non-AF: 220) as described in Section 2.1. Each patient contributed one binary image using the averaged waveforms, and no dominant waveforms were used as the same for internal testing. The age distribution of patients was analyzed and compared with that of the training and validation datasets, as shown in Table 1. Notably, in the external testing dataset presented on the right side of Table 1, there was a 17% decrease in patients aged 40 to 59, and a 14% increase in patients aged 70 to 89, compared with the training and validation dataset. This distribution trend more closely resembled the prevalence proportion observed in the age group of the AF population, and the proportion of female patients was observed to be a 5% increase in the external testing dataset as well. Additionally, in addition to collecting ECGs from patients visiting the Department of Cardiovascular Medicine for training, validation, and internal testing datasets, we also included ECGs from patients transported by emergency or other departments in the external testing dataset. This broader sample allows for a more comprehensive validation of the generalization ability of the developed AI model. We acknowledge that conducting further analysis to assess the impact of age and gender-related physiological changes on model performance would be beneficial. To address this, we plan to collect additional data with a balanced distribution of patients across age groups and genders in the future.

#### 3.2.2. Additional Measures of Bias Minimization

To minimize the influence of bias from patient characteristics between AF and non-AF groups on performance evaluation, additional steps were taken. After selecting the necessary ECGs of AF patients randomly from the available data, an equal number of non-AF ECGs were selected. These selections were not only matched with the clinical characteristics but also the age distribution of the patients in the AF group. The results, as presented in Table 2, indicate that apart from a higher number of diabetes patients in the non-AF group compared to the AF group, other patient characteristic items were quite similar between the two groups. Furthermore, patients with both normal (borderline-normal included) and abnormal (borderline-abnormal included) ECGs during ECG automatic interpretation were well balanced as well. Therefore, no bias effect existed during the external performance evaluation. This approach helps ensure the robustness and reliability of the model’s performance evaluation process by mitigating potential biases. However, the disparity in the occurrence of diabetes, similar to that observed in the training dataset, may be attributed to patients without a history of AF predominantly visiting the hospital for periodic inspections. Additionally, information on whether patients were hospitalized was not recorded during the data collection. This may potentially introduce selection bias. We plan to mitigate this limitation by including this information in future investigations.

#### 3.2.3. Statistical Analysis of Patient Characteristics

According to the first step of the statistical analysis plan outlined in Section 2.6, patient characteristics were statistically analyzed. The results are summarized in Table 3. It was observed that several data points were missing in the smoking and hypertension items. The mean values of age, height, and weight for patients in both groups were approximately 70 years, 160 cm, and 61 kg, respectively. Additionally, approximately 61% of patients were male.

The *p*-values for the continuous variables and categorical variables were obtained from the F-test, student *t*-tests, and Fisher exact tests, respectively. With the exception of the diabetes item, all *p*-values were greater than the 0.05 significance level. This indicates that there were no statistically significant differences in clinical characteristics between patients in the AF and the non-AF groups.

#### 3.2.4. Performance Validation

In the second step of the statistical analysis plan, the performance of the fitted model used for external validation was assessed. The following performance metrics were evaluated: AUC, 0.80 (95% CI 0.76–0.84); sensitivity, 72.3% (95% CI 65.9–78.1); specificity, 77.7% (95% CI 71.6–83.0); precision, 76.4% (95% CI 70.1–82.0); and accuracy, 75.0% (95% CI 70.7–79.0), respectively. Two-sided 95% confidence intervals for the measured metrics were estimated with the Delong method for AUC and the Clopper–Pearson method for the other metrics. The ROC curve obtained from the external testing is depicted on the right side of Figure 5b shown in Section 3.1 to compare with the ROC curve in Figure 5a obtained from the internal testing. The mere 2% difference compared to the AUC from the internal testing suggests a strong generalizability of the fitted model.

#### 3.2.5. Special Non-AF Data Testing

To minimize the risk of mislabeling patients in the non-AF group who may have undetected AF, several measures were implemented. Patients presenting with a chief complaint of palpitations or subjective symptoms were excluded from the non-AF data collection. Moreover, at least two SR ECGs recorded in the past five years based on the latest selected SR ECG were required for inclusion. In the third step of the statistical analysis plan, patients with palpitations but diagnosed with inappropriate sinus tachycardia (IST), or atrioventricular nodal reentrant tachycardia (AVNRT) after catheter ablation had their SR ECGs collected before the catheter ablation and utilized as special data for non-AF identification.

Data from a total of 29 patients for this special non-AF dataset were collected, comprising 12 males and 17 females, with ages ranging from 40 to 80. The detection rate of non-AF in this subset was 75.9% (95% CI 56.5–89.7), which was slightly lower (1.8%) than the specificity of 77.7% measured from the external testing data (*n* = 440). This outcome further indicates a successful detection rate on the special data with similar accuracy for non-AF identification.

Each patient contributed one binary image using the averaged waveforms, ensuring no data duplication occurred in the special data testing. Furthermore, we utilized the arrhythmia exclusion list described in Section 2.2 to exclude patients with heart diseases unrelated to AF.

### 3.3. Successful Deployment

The app package with the edge AI model was successfully deployed on an edge ECG device, where the time for detecting AF risk on-board was measured to be approximately 2 s, nearly in real time following an automated diagnosis of routine standard 10 s, 12-lead ECGs. Additionally, the prediction results obtained on the edge device after deployment were confirmed to be completely the same compared with the results predicted on a PC in a Python environment. These comparative results validated the low-cost and successful deployment of the method.

## 4. Discussion

### 4.1. Major Findings and Key Outcomes

1. Existing screening methods for AF often miss cases due to the condition’s paroxysmal and asymptomatic nature. This under-detection can lead to serious consequences such as stroke and premature death. The findings of this study highlight the potential of deep learning-based edge AI models in the early detection of AF during normal SR using standard 10 s, 12-lead ECGs.
2. The inclusion and exclusion criteria for data collection, such as age over 40 years old, the presence of normal SR, and the exclusion of the ECGs included in a defined arrhythmia exclusion list, aimed to capture a representative sample of patients who may have had undetected AF in the past 2 years but are not currently experiencing symptomatic episodes.
3. The well-balanced data collection, with each patient contributing an equal amount of ECG data, specifically one ECG datum per patient for testing; the additional measures of bias minimization in the two groups; and the rigorous labeling process conducted by trained physicians under cardiologist supervision, ensured the reliability of the datasets for model training, testing, and accurate performance evaluation.
4. The proposed method involved converting the averaged waveform from each lead of a standard 10 s, 12-lead ECG into a binary image and then composing them. This approach facilitated the training of a lightweight CNN model for AF risk detection during SR.
5. The performance metrics of the deployed model, including sensitivity, specificity, precision, overall accuracy, and AUC, demonstrate its effectiveness and generalization capability in detecting AF risk during SR in both internal and external testing datasets.
6. The model maintains comparable performance and improves its suitability for deployment on resource-constrained devices, thereby expanding its potential impact to a wide range of healthcare settings. Its successful deployment enables real-time AF risk detection during SR in clinical settings where immediate intervention is crucial.

### 4.2. AUC and Methods Comparison

The estimated AUCs for the fitted model in internal and external testing were 0.82 (95% CI: 0.77–0.88) and 0.80 (95% CI: 0.76–0.84), respectively. These values outperform those of other medical screening tests, such as B-type natriuretic peptide (BNP) for heart failure and cardiovascular disease diagnosis (AUC: 0.60–0.70) [12], Papanicolaou smear for cervical cancer screening (AUC: 0.70) [13], and the CHA2DS2-VASc score for stroke risk assessment (AUC: 0.57–0.72) [14].

Several studies have been reported for AF risk detection during SR ECGs in the last 5 years. The differences between this study and others are summarized in Table 4.

We acknowledge that a broader comparison with other models tested on similar external datasets would enhance the understanding of our model’s performance within the field. However, currently, we do not have access to other existing models tested on the same external dataset. We will continue to explore opportunities to conduct such comparisons in future work.

The achieved sensitivity and specificity of the external testing were balanced at the optimized cutoff of 0.467. This threshold can be adjusted depending on clinical needs. A low cutoff with high sensitivity may be useful in excluding healthy individuals who do not require further inspection, while a high cutoff with high specificity may be beneficial for identifying patients with a high pretest probability for intensive monitoring.

By employing the R wave-triggered signal averaging method to generate averaged waveforms from SR ECGs and then converting them into binary images, a lightweight CNN model was trained. This approach proved to be efficient and feasible for AF risk detection on resource-constrained ECG devices, with an approximate time of 2 s after automatic 12-lead ECG interpretation.

### 4.3. Limitations and Future Directions

Several limitations were identified in this study. First, all ECG data were retrospectively collected from general or university-affiliated hospitals, necessitating further evaluation in a broader, ostensibly healthy population. Second, although a total of 1502 AF-labeled ECGs, with one datum per patient, were collected, which is more than some other studies, the relatively moderate scale of the ECG dataset for AI model training may limit model performance and robustness. This warrants further analysis with additional data. Finally, being a multi-center retrospective study, prospective, large-scale studies are required to validate the model’s performance in the future.

## 5. Conclusions

The proposed method, which involved extracting averaged waveforms from standard 10 s, 12-lead SR ECGs and converting them into binary images, facilitated the training of a lightweight CNN model for AF risk detection during SR. The achieved performance, as evaluated from internal and external datasets, demonstrated the effectiveness and generalization capability of the trained model in detecting undiagnosed AF.

Moreover, the successful deployment of the app package on edge ECG devices enables the practical application of undiagnosed AF detection in real time during SR. This development marks a significant contribution to the advancement of AI in healthcare and holds important implications for early AF screening and the management of patients with unexplained strokes.

Moving forward, further improvements can be explored through the utilization of large-scale data. Continual refinement and validation of the model’s performance will be essential for its continued effectiveness and reliability in clinical practice.

Overall, the deployment of the model on edge AI ECG devices represents a significant step towards enhancing healthcare outcomes and addressing the challenges associated with undiagnosed AF, ultimately improving patient care and management strategies.

## Figures and Tables

**Table 1:** Age distribution comparison between training and external testing datasets.

Age Group	Training + Validation (*n* = 2330)				External Testing (*n* = 440)			
	Patients	Female	Male	Proportion	Patients	Female	Male	Proportion
40–49	329	101	228	14.1%	19	6	13	4.3%
50–59	438	115	323	18.8%	52	16	36	11.8%
60–69	628	192	436	27.0%	125	50	75	28.4%
70–79	673	275	398	28.9%	162	67	95	36.8%
80–89	250	103	147	10.7%	74	31	43	16.8%
90–100	12	5	7	0.5%	8	2	6	1.8%
Total	2330	791	1539	100.0%	440	172	268	100.0%
Proportion		33.9%	66.1%	100.0%		39.1%	60.9%	100.0%

**Table 2:** Matched patient characteristics in two groups for external testing.

Items	Non-AF	AF	Items	Non-AF	AF
40–49	10	9	Male	134	134
50–59	26	26	Female	86	86
60–69	63	62	Smoking	118	118
70–79	80	82	Hypertension	118	115
80–89	37	37	Diabetes	78	47
90–99	4	4	Normal (ECGs)	121	122
Total	220	220	Abnormal (ECGs)	99	98

**Table 3:** Statistical analysis of the patient characteristics in the external testing dataset.

Items	Non-AF Group *n* = 220	AF Group *n* = 220	*p*-Value of F-Test	*p*-Value of *t*-Test	*p*-Value of Fisher Exact Test
Age	69.8 ± 10.7	70.3 ± 10.8	0.848	0.638	NA
Height	160.1 ± 9.5	160.3 ± 10.3	0.218	0.846	NA
Weight	60.9 ± 14.5	61.5 ± 14.6	0.880	0.700	NA
BMI	23.7 ± 5.1	23.8 ± 5.1	0.935	0.802	NA
Gender (M/F)	134/86	134/86	NA	NA	1.000
Smoking (NaN/F/T)	1/101/118	1/101/118	NA	NA	1.000
Hypertension (NaN/F/T)	0/102/118	3/102/115	NA	NA	0.324
Diabetes (F/T)	142/78	173/47	NA	NA	0.001

NaN: Missing data; F: False; T: True. NA: Not Available.

**Table 4:** Method and performance comparison between this study and the other studies.

Items	The Other Studies	This Study
Age of patients	18 years or older [3,5,6]	40 years or older
Training and testing dataset	Imbalanced [3,5]	Well balanced
Testing data	Multiple ECGs per patient [3,5,6]	One ECG data per patient
Bias minimization	Unreported [3,5,6]	Conducted for external testing
Model input type	Time-series ECGs with multiple leads (8 or 12), each lasting 8 or 10 s [3,5,6]	Binary ECG images with 12-lead averaged waveforms
AI model	Resnet [3,6], RNN [6], LSTM [5]	Standard CNN
AUC from internal testing	0.87 [3], 0.79 [5,6]	0.82
AUC from external testing	0.75 [5]	0.80
Type of product	Algorithm [3,5,6]	Edge AI deployed

## Data Availability

The datasets used for training, validation, and internal testing were provided by AMED. Information regarding the data management guidelines and access platform can be found at the following link: https://www.amed.go.jp/koubo/datamanagement.html (accessed on 23 March 2024). While the construction of the platform for data reuse is ongoing, researchers can visit the link to check its progress and submit requests for dataset access. External testing data were collected by Fukuda Denshi, Tokyo, Japan, with consent obtained through an opt-out form on a website. As participants were informed that the data would be used solely for this study, we regret that it cannot be shared publicly.

## Supplementary Materials

The following supporting information can be downloaded at https://www.mdpi.com/article/10.3390/jcm13082218/s1: Table S1: The arrhythmia exclusion list.

## References

[1] Haim M., Hoshen M., Reges O., Rabi Y., Balicer R., Leibowitz M. (2015). Prospective national study of the prevalence, incidence, management and outcome of a large contemporary cohort of patients with incident non-valvular atrial fibrillation. J. Am. Heart Assoc..

[2] Wolf P.A., Abbott R.D., Kannel W.B. (1991). Atrial fibrillation as an independent risk factor for stroke: The Framingham study. Stroke.

[3] Attia Z.I., Noseworthy P.A., Lopez-Jimenez F., Asirvatham S.J., Deshmukh A.J., Gersh B.J., Carter R.E., Yao X., Rabinstein A.A., Erickson B.J. (2019). An artificial intelligence-enabled ECG algorithm for the identification of patients with atrial fibrillation during sinus rhythm: A retrospective analysis of outcome prediction. Lancet.

[4] Somani S., Russak A.J., Richter F., Zhao S., Vaid A., Chaudhry F., De Freitas J.K., Naik N., Miotto R., Nadkarni G.N. (2021). Deep learning and the electrocardiogram: Review of the current state-of-the-art. Europace.

[5] Baek Y., Lee S., Choi W., Kim D. (2021). A new deep learning algorithm of 12-lead electrocardiogram for identifying atrial fibrillation during sinus rhythm. Sci. Rep..

[6] Melzi P., Tolosana R., Cecconi A., Sanz-Garcia A., Ortega G.J., Jimenez-Borreguero L.J., Vera-Rodriguez R. (2021). Analyzing artificial intelligence systems for the prediction of atrial fibrillation from sinus-rhythm ECGs including demographics and feature visualization. Sci. Rep..

[7] Jeni L.A., Cohn J.F., Torre F.D.L. Facing Imbalanced Data Recommendations for the Use of Performance Metrics. Proceedings of the 2013 Humaine Association Conference on Affective Computing and Intelligent Interaction.

[8] Brownlee J. (2020). Imbalanced Classification with Python.

[9] Murat F., Sadak F., Yildirim O., Talo M., Murat E., Karabatak M., Demir Y., Tan R., Acharya R. (2021). Review of Deep Learning-Based Atrial Fibrillation Detection Studies. Int. J. Environ. Res. Public Health.

[10] Inoue H., Fujiki A., Origasa H., Ogawa S., Okumura K., Kubota I., Aizawa Y., Yamashita T., Atarashi H., Horie M. (2009). Prevalence of atrial fibrillation in the general population of Japan: An analysis based on periodic health examination. Int. J. Cardiol..

[11] Iguchi Y., Kimura K., Aoki J., Kobayashi K., Terasawa Y., Sakai K., Shibazaki K. (2008). Prevalence of Atrial Fibrillation in Community-Dwelling Japanese Aged 40 Years or Older in Japan. Circ. J..

[12] Bhalla V., Isakson S., Bhalla M.A., Lin J.P., Clopton P., Gardetto N., Maisel A.S. (2005). Diagnostic ability of B-type natriuretic peptide and impedance cardiography: Testing to identify left ventricular dysfunction in hypertensive patients. Am. J. Hypertens..

[13] Chen Y., Cui Z., Xiao Z., Hu M., Jiang C., Lin Y., Chen Y. (2016). PAX1 and SOX1 methylation as an initial screening method for cervical cancer: A meta-analysis of individual studies in Asians. Ann. Transl. Med..

[14] Wu J., Wang S., Chu Y., Long D., Dong J., Fan X., Yang H., Duan H., Yan L., Qian P. (2017). CHADS2 and CHA2DS2-VASc scores predict the risk of ischemic stroke outcome in patients with interatrial block without atrial fibrillation. J. Atheroscler. Thromb..

