# Supplementary material for: Edge AI Model Deployed for Real-Time Detection of Atrial Fibrillation Risk during Sinus Rhythm
Source: J Clin Med. 2024 Apr 11;13(8):2218. doi: 10.3390/jcm13082218 (PMC11051059; doi:10.3390/jcm13082218)
Supplement: Supplementary file 1 [file jcm-13-02218-s001.zip › jcm-2956270-supplementary.pdf]

Table S1. The arrhythmia exclusion list.

Second-degree Atrioventricular Block (Wenckebach)	PAC Trigeminy
Complete Atrioventricular Block	PVC Trigeminy
Second-degree Atrioventricular Block (Mobitz)	Frequent PAC
Second-degree Atrioventricular (2:1)	Frequent PVC
Artificial Pacemaker Rhythm	PAC Bigeminy
Suspected Left Atrial Rhythm	PVC Bigeminy
Atrioventricular Junctional Rhythm	Sinoatrial (S-A) Block
Atrioventricular Dissociation	Supraventricular Tachycardia
Tachycardia	Short Runs of PAC
Bradycardia	Ventricular Tachycardia
Extreme Tachycardia	Short Runs of PVC
Extreme Bradycardia	Ventricular Rhythm
Escape Beat	Couplet PAC
Premature Atrial Contraction (PAC)	Couplet PVC
Premature Ventricular Contraction (PVC)	Atrial Fibrillation and Flutter
